# Divergent Leaf Morpho-Physiological and Anatomical Adaptations of Four Lettuce Cultivars in Response to Different Greenhouse Irradiance Levels in Early Summer Season

**DOI:** 10.3390/plants10061179

**Published:** 2021-06-09

**Authors:** Luigi Formisano, Michele Ciriello, Valerio Cirillo, Antonio Pannico, Christophe El-Nakhel, Francesco Cristofano, Luigi Giuseppe Duri, Maria Giordano, Youssef Rouphael, Stefania De Pascale

**Affiliations:** Department of Agricultural Sciences, University of Naples Federico II, 80055 Portici, Italy; luigi.formisano3@unina.it (L.F.); michele.ciriello@unina.it (M.C.); valerio.cirillo@unina.it (V.C.); antonio.pannico@unina.it (A.P.); christophe.elnakhel@unina.it (C.E.-N.); francesco.cristofano@unina.it (F.C.); luigigiuseppe.duri@unina.it (L.G.D.); maria.giordano@unina.it (M.G.)

**Keywords:** *Lactuca sativa* L., sub-optimal conditions, greenhouse, leaf gas exchange, F_v_/F_m_ ratio, LMA, stomata

## Abstract

Lettuce (*Lactuca sativa* L.) is a winter-spring leafy vegetable, but the high demand for fresh products available year-round requires off-season production. However, the warm climate of the Mediterranean areas can impair the summer production of lettuce, thus requiring the adoption of genotypes tolerant to high irradiance as well as useful agronomic strategies like shading net installations. The aim of our research was to assess the leaf morpho-physiological and anatomical changes, in addition to productive responses, of four lettuce cultivars (‘Ballerina’, ‘Maravilla De Verano Canasta’, ‘Opalix’, and ‘Integral’) grown under shading and non-shading conditions to unveil the adaptive mechanisms of this crop in response to sub-optimal microclimate (high irradiance and temperature) in a protected environment. Growth and yield parameters, leaf gas exchanges, chlorophyll fluorescence and morpho-anatomical leaf traits (i.e., leaf mass area, stomatal density and epidermal cell density) were determined. Under shading conditions, the fresh yields of the cultivars ‘Ballerina’, ‘Opalix’ (‘Oak leaf’) and ‘Integral’ (‘Romaine’) increased by 16.0%, 26.9% and 13.2% respectively, compared to non-shading conditions while both abaxial and adaxial stomatal density decreased. In contrast, ‘Canasta’ under non-shading conditions increased fresh yield, dry biomass and instantaneous water use efficiency by 9.6%, 18.0% and 15.7%, respectively, while reduced abaxial stomatal density by 30.4%, compared to shading conditions. Regardless of cultivar, the unshaded treatment increased the leaf mass area by 19.5%. Even though high light intensity and high temperature are critical limiting factors for summer lettuce cultivation in a protected environment, ‘Canasta’ showed the most effective adaptive mechanisms and had the best production performance under sub-optimal microclimatic conditions. However, greenhouse coverage with a white shading net (49% screening) proved to be a suitable agricultural practice that ensured an adequate microclimate for the off-season growth of more sensitive cultivars ‘Ballerina’, ‘Oak leaf’ and ‘Romaine’.

## 1. Introduction

Lettuce (*Asteraceae; Lactuca sativa* L.) is one of the most used and popular leafy vegetables globally, but its nutritional value is underestimated for its high water content (about 95%) [1,2]. Lettuce is an essential source of minerals (e.g., potassium, calcium, phosphorus, magnesium, iron and zinc), which help maintain the correct hydro-saline balance of the human body, other than being rich in fibers, bioactive compounds, vitamins and carotenoids that are beneficial molecules for the human health [3,4]. Being a species adapted to low temperatures and low light intensity, lettuce is generally grown in winter and spring seasons. However, the high demand for year-round products has led to off-season cultivation of lettuce (spring-summer) in protected environments [5]. Extending lettuce cultivation into off-season production, where the demand for fresh products is higher, ensures growers better prices with significant economic benefits [6].

High temperatures and high irradiance are typical of Mediterranean summers; such conditions are a limiting factor for agriculture, especially in sensitive crops such as lettuce, as they lead to morpho-physiological alterations that induce crop yield losses and quality impairments (e.g., head closure, rib discoloration, tipburn) [7,8,9,10,11]. Considering that the optimal temperatures for lettuce growth range from 18 to 28 °C, high-temperature stress combined with a long day induces alterations in water relations, photosynthetic activity, osmolyte accumulation and hormone production [12,13]. Other than leading to quality degradation [14], these changes lead to a lower marketable yield of lettuce, which is affected by dry matter and water content [15]. To avoid these adverse effects, off-season lettuce production requires adequate crop protection from high solar radiation. In this perspective, shading nets, due to their ability to reduce light intensity, modulate light diffusion and hence reduce temperature, are effective at extending the growing season and improving the quality of horticultural products [16,17]. During summer, shading nets are widely used in Mediterranean areas to create a suitable microclimate for crop production, consequently reducing photoinhibition and improving water use efficiency and crop uniformity [16,18].

Light fosters lettuce growth only in a specific range of light intensities [19]. Several studies have shown that lettuce grown in summer with light intensity over 600 μmol m^−2^ s^−1^ had reduced biomass, leaf area and chlorophyll content [19,20]. These reductions were mainly ascribed to a low instantaneous saturation point, with evidence of oxidative processes (photoinhibition) at 800 μmol m^−2^ s^−1^, as reflected by the lower F_v_/F_m_ values compared to other plants that would grow well at levels of light intensity higher than 600 μmol m^−2^ s^−1^ [5]. To cope with oxidative damage under high-light stress, plants have evolved complex adaptive mechanisms, including short and long-term responses [21,22]. Within hours of the stressful event, plants reduce their photosynthetic activity by closing the stomata, changing the orientation of leaves (heliotropism) and rearranging chloroplasts parallel to the light direction (avoidance response) [21,23,24,25,26]. In the long-term, light stress triggers morpho-physiological changes in the plant, such as a reduction in chlorophyll content and leaf area and an increase in leaf mass area (LMA) [23,27,28]. As observed by Zha et al. [29] in *Lactuca sativa* L., small and thick leaves (higher LMA) have better adaptability to high light intensity by reducing water loss and improving light utilization. Several authors have reported that smaller and thicker leaves show improved heat exchange efficiency, which prevents rapid temperature rise and the consequent water loss under high light conditions [30,31,32,33]. Moreover, higher biomass investment in the leaf, as generally found in thicker leaves with higher LMA, has been correlated with an enhanced photosynthetic capacity [28]. However, the morpho-physiological response to light intensity may differ among cultivars due to their genetic background [28].

In response to external stimuli, plants also change the density and size of stomata to ensure a rapid improvement of water use efficiency under sub-optimal growth conditions [34,35,36,37]. In general, high light triggers stomatal development [38], while heat stress has an opposite effect [39,40]. Summer cultivation in the Mediterranean environment couples the effects of excessive light and heat with a detrimental effect on the productivity of not suited crops, such as lettuce. The high demand for evapotranspiration that characterizes this environment implies that the balance between water loss and leaf cooling is a key aspect for plants to thrive, which is partially mediated by the plasticity of stomatal patterning [41]. Muir [42] has observed that high light intensity increased the adaxial stomatal density, which is more exposed to heating, to prevent harmful water loss [38]. The alteration of the stomatal density also impacts the plant’s growth rate [35]. However, under the same climatic conditions, water use efficiency shows considerable intraspecific variability [43]. Indeed, plant adaptation to sub-optimal conditions depends on the genotype, environment and their mutual interaction [14]. Several studies have shown that lettuce cultivars with red leaves have better tolerance to high solar radiation than cultivars with green ones, which are more susceptible to photooxidation [44,45]. The high anthocyanin content of red cultivars would probably act as an antioxidant, shielding solar radiation and leading to better adaptability to high light conditions [44,46]. The high genetic variability of lettuce represents an important resource for studying the responses of this crop to different environmental constraints, which will enable conscious breeding programs focused on increasing its adaptability in the modern climate change scenario [11].

The genetic variability in leaf morphology and pigmentation combined with the most advanced shading technology could be exploited to extend the growing season of lettuce in regions where high light intensity and high temperatures are limiting factors. For this purpose, the morpho-physiological and productive responses of four lettuce cultivars ordinarily grown in open field (‘Ballerina’, ‘Canasta’, ‘Oak leaf’ and ‘Romaine’) were evaluated under shading and non-shading conditions to identify the most suitable genotype for cultivation under sub-optimal early summer conditions in a passively ventilated greenhouse. Even though lettuce is one of the most globally consumed vegetables, its susceptibility to the extreme environmental conditions of warm Mediterranean areas severely limits its off-season cultivation. To date, few studies have focused on the adaptive mechanisms of lettuce grown under suboptimal microclimatic conditions like summer greenhouse cultivation. Based on these considerations, it is interesting to understand how different lettuce cultivars respond to extreme conditions in both shaded and unshaded greenhouses by activating specific adaptive mechanisms. As far as we know, this is the first research investigating these aspects, and our results could be useful for both growers and breeders, paving the way for future work.

## 2. Results

### 2.1. Biometric and Yield Parameters in Response to Different Greenhouse Irradiance Conditions

As shown in Table 1 all biometric and yield parameters were affected by the interaction between cultivar (CV) and greenhouse irradiance conditions (GIC) factors. Regarding the leaf number, greenhouse irradiance conditions did not result in a univocal response among cultivars. Specifically, for ‘Canasta’ and ‘Romaine’ was observed a reduction in the leaf number under the shading net by 7.7% and 16.8%. In contrast, the shading net increases this parameter (leaf number) in ‘Ballerina’ and ‘Oak leaf’ by 6.5% and 8.5%, respectively. Leaf area and fresh yield increased in all cultivars grown under the shading net, except ‘Canasta’ for which these parameters did not change vs. non-shading conditions. Particularly, ‘Ballerina’, ‘Oak leaf’ and ‘Romaine’ increased leaf area by 14.8%, 58.7% and 18.2% and fresh yield by 16.0%, 26.9% and 13.2%, respectively. In contrast, ‘Canasta’ recorded the highest fresh yield (285.7 g plant^−1^) in the unshaded treatment. With respect to dry biomass, both shaded and unshaded treatment did not result in any significant difference in all cultivars. In contrast, ‘Canasta’ showed a 15.2% reduction of dry biomass under the shading net. Finally, under shading ‘Ballerina’, ‘Canasta’, ‘Oak leaf’ and ‘Romaine’ decreased leaf dry matter by 14.9%, 7.1%, 16.1% and 10.8%, respectively.

### 2.2. Macronutrients Accumulation in Response to Greenhouse Irradiance Conditions

As observed for the biometric parameters, total nitrogen, nitrate and macronutrient contents were affected by the CV × GIC interaction (Table 2). Except for ‘Romaine’, the total nitrogen concentration in the leaves of ‘Ballerina’, ‘Canasta’ and ‘Oak leaf’ increased under the shading net by 13.1%, 9.7% and 14.7%, respectively. The same trend was observed for nitrate content which increased under shading net for all cultivars, except for ‘Romaine’. Notably, the highest increase in nitrate was recorded in ‘Ballerina’ (+14.9%). For all cultivars, there was a significant increase in phosphorus content when the shading net was used. The same trend was observed for potassium in ‘Romaine’ and ‘Oak leaf’, which increased by 13.5% and 32.1%, respectively, while for ‘Ballerina’ and ‘Canasta’, GIC treatment did not affect potassium build-up. ‘Oak leaf’ showed a significant increase in sodium (23.8%) and magnesium (44.0%) in the shaded treatment compared to the unshaded one. In contrast, the unshaded treatment increased calcium content by 35.2%, 83.5%, 16.7% and 24.1% in ‘Ballerina’, ‘Canasta’, ‘Oak leaf’ and ‘Romaine’, respectively. On the other hand, sulfur content increased in all cultivars except for ‘Oak leaf’ in the unshaded treatment.

### 2.3. SPAD Index, Chlorophyll Fluorescence Emission and Leaf Mass Area (LMA) in Response to Greenhouse Irradiance Conditions

As reported in Table 3, the SPAD index measured at different days after transplant (8, 14 and 21 DAT) were affected by the CV × GIC interaction. At 8 DAT, all cultivars showed SPAD index reduction in the shaded treatment. At 14 DAT, the same trend was observed only for ‘Canasta’ and ‘Oak leaf’. Moreover, at 21 DAT, the highest SPAD index values were recorded in ‘Ballerina’ (36.77) in shaded treatment and ‘Oak leaf’ (24.82) in unshaded treatment, whereas the other two cultivars showed no significant difference between shaded and unshaded treatments.

Fluorescence and leaf mass area (LMA) values showed significant differences only for the means values of both factors (CV and GIC) (Table 3). Regardless of the cultivar, shading net increased the F_v_/F_m_ ratio by 10.7% and reduced the LMA by 19.6%. The latter parameter showed significant cultivar-dependent response (‘Ballerina’ > ‘Romaine’ > ‘Canasta’ > ‘Oak leaf’).

### 2.4. Instantaneous Water Use Efficiency and Morpho-Anatomical Leaf Traits in Response to Greenhouse Irradiance Conditions

**Figure 1:**
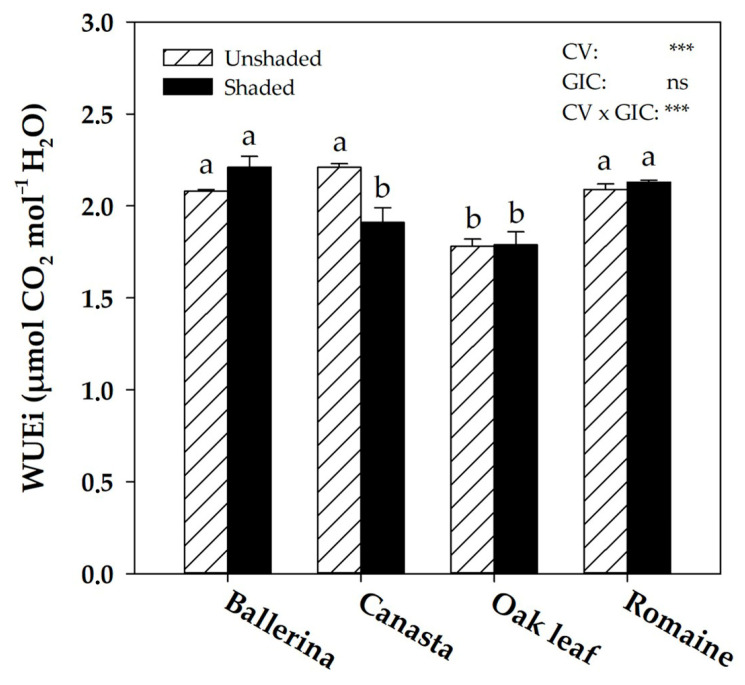
Effect of Cultivar (CV) and Greenhouse Irradiance Conditions (GIC) on instantaneous water use efficiency (WUEi) in *Lactuca sativa* L. Data are mean values ± standard error, *n* = 3. Mean comparisons were performed by Duncan’s Multiple Range Test (DMRT) for CV and by *t*-Test for GIC. Different letters indicate significant differences compared by DMRT (*p* = 0.05). ns and *** denote nonsignificant or significant effect at *p* ≤ 0.001, respectively.

The CV × GIC interaction did not result in any variation in leaf gas exchanges (A_CO2_, g_s_ and E), which were affected exclusively by the mean cultivar effect (data not shown). In contrast, the instantaneous water use efficiency (WUEi) was affected by the CV × GIC interaction, where the difference was only significant in ‘Canasta’, +15.7% in the unshaded treatment in comparison to shaded (Figure 1).

**Figure 2:**
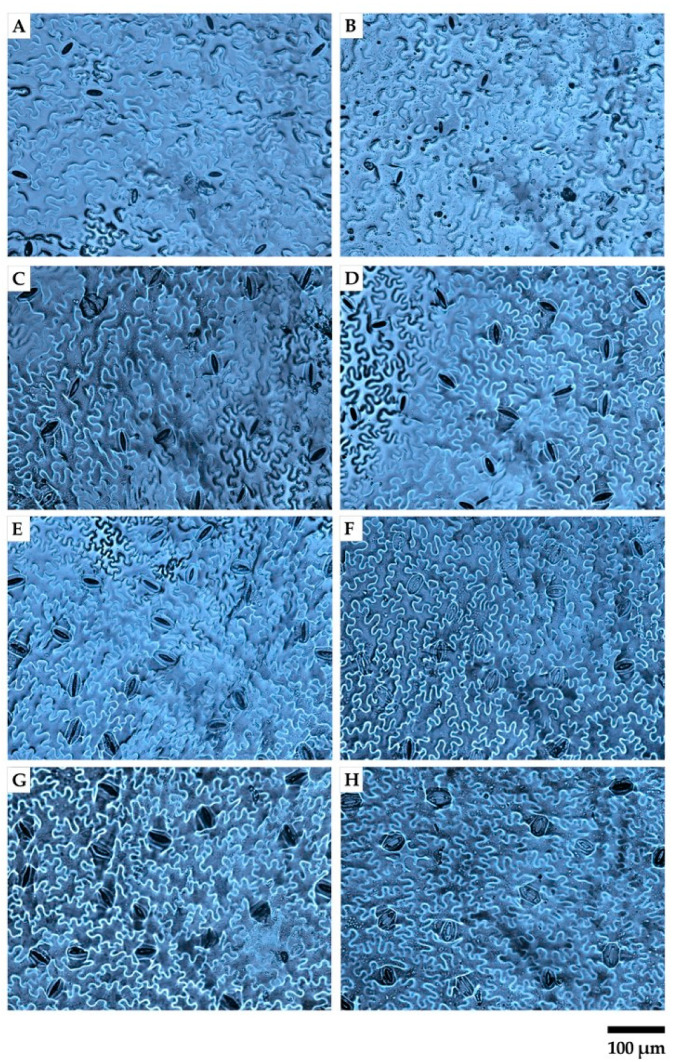
Illustrative microscopy images of the abaxial side of *Lactuca sativa* L. leaves in shaded and unshaded treatment (20×). Ballerina unshaded (**A**) and shaded (**B**); Canasta unshaded (**C**) and shaded (**D**); Oak leaf unshaded (**E**) and shaded (**F**); Romaine unshaded (**G**) and shaded (**H**).

**Figure 3:**
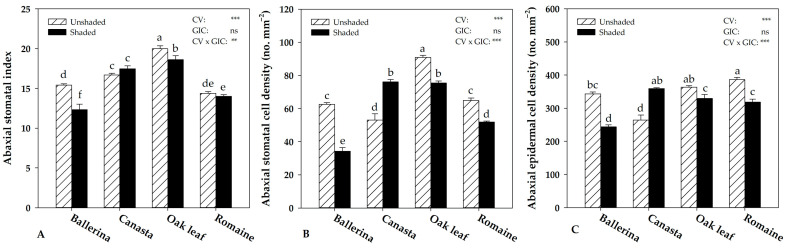
Effect of Cultivar (CV) and Greenhouse Irradiance Conditions (GIC) on morpho-anatomical traits of abaxial side of *Lactuca sativa* L. Stomatal index (**A**), stomatal cell density (**B**), and epidermal cell density (**C**). Data are mean values ± standard error, *n* = 3. Mean comparisons were performed by Duncan’s Multiple Range Test (DMRT) for CV and by *t*-Test for GIC. Different letters indicate significant differences compared by DMRT (*p* = 0.05). ns, **, and *** denote nonsignificant or significant effect at *p* ≤ 0.01 and 0.001, respectively.

Figure 2 shows illustrative microscopy images of the abaxial side of lettuce leaves in the shaded and unshaded treatment for each cultivar. Morpho-anatomical leaf traits (i.e., stomatal cell density, undulated epidermal cell density and stomatal index of abaxial and adaxial side of leaves) were affected by the interaction CV × GIC (Figure 3).

On the abaxial side of the leaves of ‘Ballerina’, ‘Oak leaf’ and ‘Romaine’, shaded treatment led to a significant reduction in stomatal and epidermal cell density while the opposite trend was observed in ‘Canasta’ (Figure 3B,C). In contrast, the stomatal index decreased in shaded treatment for ‘Ballerina’ and ‘Oak leaf’ by 20% and 6.7%, respectively, while no significant effect was found for this parameter in ‘Canasta’ and ‘Romaine’ (Figure 3A).

**Figure 4:**
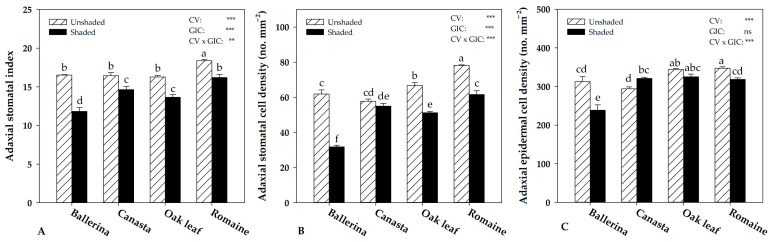
Effect of Cultivar (CV) and Greenhouse Irradiance Conditions (GIC) on morpho-anatomical traits of adaxial side of *Lactuca sativa* L. Stomatal index (**A**), stomatal cell density (**B**), and epidermal cell density (**C**). Data are mean values ± standard error, *n* = 3. Mean comparisons were performed by Duncan’s Multiple Range Test (DMRT) for CV and by *t*-Test for GIC. Different letters within columns indicate significant mean differences compared by DMRT (*p* = 0.05). ns, **, and *** denote nonsignificant or significant effects at *p* ≤ 0.01 and 0.001, respectively.

Regarding the leaves’ adaxial side, except for ‘Canasta’, all cultivars showed the highest stomatal cell density in the unshaded treatment (Figure 4B). In addition, ‘Ballerina’ and ‘Romaine’ increased epidermal cell density when cultivated without shading nets (Figure 4C). The latter parameter increased in ‘Canasta’ by 9% in the shaded treatment, while no significant effect was observed in ‘Oak leaf’. Shading net application (shaded treatment) resulted in the lowest stomatal index for all cultivars compared to the unshaded treatment (Figure 4A).

### 2.5. Leaf Pigments and Total Ascorbic Acid Accumulation in Response to Greenhouse Irradiance Conditions

As shown in Table 4, the CV × GIC interaction resulted in differences in chlorophyll and carotenoid content. Regardless of greenhouse irradiance conditions, chlorophyll *a*, *b*, total and carotenoid contents for ‘Oak leaf’ and ‘Romaine’ were unchanged. Chlorophyll *a* and total chlorophyll content in ‘Ballerina’ increased by 15.69% and 14.38%, respectively, under shaded conditions. In contrast, under the same irradiance conditions (shaded) chlorophyll *b* and total chlorophyll content in ‘Canasta’ decreased by 28.00% and 16.72%, respectively. For both cultivars (‘Ballerina’ and ‘Canasta’), carotenoid content increased when grown under shaded conditions (Table 4).

Relative to total ascorbic acid, the cultivar ‘Ballerina’ recorded a 36.16% increase when grown under shading net whereas ‘Canasta’, ‘Oak leaf’, and ‘Romaine’ exhibited no significant difference between treatments (Table 4).

## 3. Discussion

### 3.1. Leaf Morpho-Anatomical Adaptations and Productivity of Lettuce under Excessive Irradiance and Heat Conditions

The present work was aimed to assess the morpho-physiological and anatomical responses of four lettuce cultivars grown during summer in a protected environment. Interestingly, varying response to the different greenhouse irradiance conditions (shaded and unshaded) was exhibited among cultivars. ‘Canasta’ showed the best production performance under unshaded conditions due to the activation of cultivar-specific adaptive mechanisms, whereas ‘Ballerina’, ‘Oak leaf’ and ‘Romaine’ were best suited to shaded treatment (Table 1).

Irradiance plays a critical role in plant growth, and light intensity above the saturation point leads to yield loss and quality degradation [5]. Confirming the results of previous studies on leafy vegetables [44,47,48,49,50], the use of shading net increased fresh yield in ‘Ballerina’, ‘Oak leaf’ and ‘Romaine’ due to the lower temperature and solar radiation intensity, thus resulting in microclimate improvements for these lettuce cultivars (400–600 μmol m^−2^ s^−1^) (Supplementary Figure S1) [5,47]. This result is attributable to a better hydration state of the shaded plants, reflected by the increase in leaf fresh weight, decrease in dry matter % and unaltered dry biomass (Table 1). The different microclimate conditions recorded between the shaded and unshaded sub-compartments of the greenhouse did not influence the water use efficiency (WUE) of these cultivars, revealing their inability to optimize water loss under high irradiance conditions (unshaded treatment) (Figure 1). Therefore, it was necessary for these cultivars to reduce leaf area to overcome the excessive evaporative demand of the unshaded condition, which accounted for the yield loss reported at the end of the growth cycle (Supplementary Figure S2). In contrast, ‘Canasta’ showed an opposite response compared to the other cultivars, improving the productive performance in unshaded treatment, probably thanks to the improved WUE, which is relevant to conserve water resources in the Mediterranean environment [43]. The different response to the unshaded condition between ‘Canasta’ and the other cultivars in terms of WUE seems to be in line with the adaptations that occurred in leaf stomatal traits. Except for ‘Canasta’, all cultivars under high light conditions (unshaded treatment) increased stomatal density both on the abaxial and the adaxial leaf side, confirming the findings reported in the literature [36,37,42,51]. Indeed, in response to changes in light intensity, mature leaves act as stress sensors and induce stomatal density changes in newly formed leaves (long-term response), allowing the plant to adapt to adverse environmental conditions [38,51,52,53].

As previously suggested [54], lower stomatal densities are beneficial for plant growth and productivity under unfavorable environmental conditions. The lettuce cultivar ’Canasta’ reduced the abaxial stomatal density, thus improving WUE and yield (Figure 3B). Considering that epidermal cell density was significantly lower in the abaxial side of ‘Canasta’ leaves under unshaded condition (Figure 3C), which indicates a higher cell expansion compared to the shaded condition, it is possible that the lower stomatal density resulted from a “dilution effect” performed by epidermal cells on stomata [55]. This was further confirmed by the unchanged stomatal index, which indicates that stomatal initiation has not been affected by the two different irradiance conditions (Figure 3A). These results, combined with the increase in leaf number, dry biomass and unchanged leaf area, suggest that ‘Canasta’, differently from other cultivars, is better adapted to high irradiance conditions (unshaded treatment). As suggested by Zhou et al. [56], the light saturation point for some lettuce cultivars could be more than 800 μmol m^−2^ s^−1^, confirming once again the high genetic variability of this species. Regardless of cultivar, unshaded plants univocally increased LMA (Table 3) as an additional adaptive response to light stress [28]. LMA (ratio of dry biomass to leaf area) is a crucial ecological trait in plant adaptation to the environment [28]. Generally, under low light conditions, plants increase leaf area to intercept more light (lower LMA). On the other hand, under high irradiance conditions, plants increase dry biomass per unit leaf area (higher LMA) to improve photosynthetic capacity [28]. In our study, the worst performance recorded by ‘Oak leaf’ (lower leaf area, fresh yield and dry biomass) was also associated with the constitutively lowest LMA, suggesting that this cultivar is not well adapted to excessive light and temperature as imposed in our experiment [28].

### 3.2. Fluorescence, Total Ascorbic Acid and Carotenoids Content of Lettuce under Excessive Irradiance and Heat Conditions

In agreement with previous studies [29], the F_v_/F_m_ ratio varied as a function of light intensity, decreasing in plants exposed to high light intensity (unshaded treatment), probably due to photoinhibition (Table 3). However, independently of the cultivar, no changes in the main physiological and yield parameters were observed, suggesting that the decrease in F_v_/F_m_ is not solely attributable to high light photoinhibition. In fact, as observed by Lichtenthaler and Burkart [23], a minor reduction of the F_v_/F_m_ ratio does not necessarily indicate the onset of photoinhibition processes, but it could be related to other mechanisms of chlorophyll fluorescence quenching, such as heat emission and the establishment of a pH gradient. It is noteworthy that unshaded treatment increased chlorophyll a, b and total chlorophyll leaf content in all cultivars, probably to prevent the onset of harmful photoinhibition damage (Table 4). Our results are not in agreement with the reviewed literature suggesting that chlorophyll content in plant leaves decreases under high light conditions due to chloroplast formation inhibition [5,23]. This highlights how morpho-physiological and anatomical adaptive mechanisms have allowed plants to adapt efficiently to high irradiance stress (unshaded treatment).

In contrast with several studies on *Lactuca sativa* L. [2,29], the total ascorbic acid content did not increase in ‘Canasta’, ‘Romaine’ and ‘Oak leaf’ in unshaded treatment. while it was significantly reduced in ‘Ballerina’. This discordance could be due to a different genotypic response of cultivars to high irradiance intensity. As well as total ascorbic acid, carotenoids content did not show a univocal response in lettuce grown under unshaded conditions. Specifically, the decreased carotenoid contents in ‘Ballerina’ and ‘Canasta’ are in agreement with Gerganova et al. [57]. In contrast, ‘Oak leaf’ and ‘Romaine’ maintained the content of this crucial bioactive molecule unchanged, probably as a defense system to high irradiance intensity, because these pigments act as photo-selective filters [58]. The current results are not in line with the findings of Rouphael et al. [59], where ‘Red Oak leaf’ and ‘Baby Romaine’ demonstrated significantly lower carotenoid concentrations when grown under lower irradiance in a controlled environment. The same authors reported that the variation of some carotenoids could be in part attributed to the head structure of the different cultivars.

### 3.3. Leaf Ions Accumulation of Lettuce under Excessive Irradiance and Heat Conditions

The dynamics driving nitrate and mineral accumulation in vegetables are complex because of their influence by the environment × genotype interaction [2]. As expected, high irradiance intensity (unshaded treatment) reduced nitrate content in ‘Ballerina’, ‘Canasta’ and ‘Oak leaf’ because nitrate reductase is more efficient at high light intensity [59]. However, the lower nitrate content could also be attributed to the improved activity of other crucial enzymes such as glutamate synthetase and glutamine synthetase and the inhibition of asparagine synthetase involved in nitrate stabilization and transport processes [60]. In addition, the same cultivars showed a negative correlation between nitrate accumulation and leaf dry matter, as pointed out by Reinink et al. [61] in *Lactuca sativa* L. It is noteworthy that ‘Romaine’ did not change in nitrate content under shaded conditions, probably due to a lower constitutive concentration dependent on genotype [2]. Similarly, total nitrogen content showed the same nitrate trend, as supported by the literature review [19]. Like nitrogen, phosphorus is a key element for plant growth and productivity, playing a pivotal role in cellular processes, membrane maintenance and energy molecules biosynthesis [62]. Our results showed a univocal response of cultivars to phosphorus accumulation, decreasing at high light intensity (unshaded treatment). Since phosphorus is essential for maintaining the photosynthetic machinery (PSII) [63], its lower values, regardless of cultivar, would be justified by the lower F_v_/F_m_ ratio obtained in unshaded plants.

In contrast, in all cultivars, leaf calcium content increased under unshaded conditions. This higher calcium accumulation could be due to plants’ lower growth rate under high light conditions (unshaded treatment), except for ‘Canasta’, which grew faster (Supplementary Figure S2). Calcium is a poorly mobile element, and therefore higher growth speed might have reduced for ‘Ballerina’, ‘Oak leaf’ and ‘Romaine’ the translocation of calcium [11]. However, in addition to maintaining membrane and cell wall structure, calcium acts as a signal molecule, promoting the activation of specific adaptive mechanisms that help plants adapt to various abiotic stresses (e.g., high light and high temperature) [64]. In our experiment, the higher concentration of calcium in unshaded plants could result from the fact that calcium had helped improve plants’ resistance under light stress. Specifically, it is interesting to note that ‘Canasta’ showed the highest calcium accumulation (+85%) under unshaded condition, indicating a better adaptation to light stress and improved production performance (greater fresh yield and dry biomass) [64].

## 4. Materials and Methods

### 4.1. Experimental Design, Plant Material and Growth Conditions

**Figure 5:**
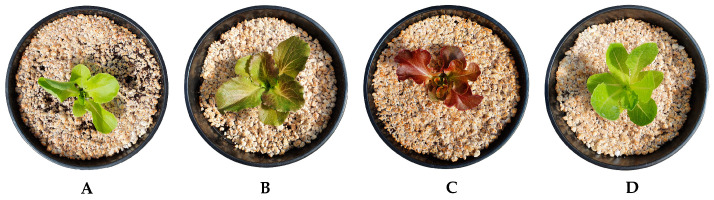
Illustrative picture of *Lactuca sativa* L. genotypes used in the experiment at transplant. ‘Ballerina’ (**A**), ‘Canasta’ (**B**), ‘Oak leaf’ (**C**), and ‘Romaine’ (**D**).

The experimental trial was conducted during the early summer season 2020 in a glass greenhouse located at the Department of Agriculture (DIA) of the University of Naples Federico II (Portici, Italy; 40°49′ N, 14°15′ E, 72 m a.s.l.). The experimental protocol included a white shading net with a 49% light screening factor (2681BL Prisma MDF; Arrigoni S.P.A, Uggiate Trevano, Como, Italy) and an unshaded treatment in factorial combination with four lettuce (*Lactuca sativa* L.) cultivars with different morphology of leaves (Figure 5). The glasshouse was split into independent compartments of 15 m length and 5 m width each, representing the shaded and unshaded treatments. Plants of each cultivar were randomized in each compartment. Each compartment contained 4 experimental units (one for each cultivar) including six plants (24 plants per compartment). Lettuce cultivars ‘Ballerina’ (Butterhead lettuce, Rijk Zwaan, De Lier, The Netherlands), ‘Maravilla De Verano Canasta’ hereafter ‘Canasta’ (Butterhead lettuce, Pagano Domenico and Figli, Scafati, Salerno, Italy), ‘Opalix’ hereafter ‘Oak leaf’ (Leaf lettuce; Enza Zaden, Enkhuizen, The Netherlands) and ‘Integral’ hereafter ‘Romaine’ (Cos lettuce; Syngenta, Basel, Switzerland) were transplanted on June 16 into pots (15 × 15 cm, 1.8 L) filled with a 2:1 substrate (*v*/*v*) of peat and perlite. The pots were covered with a fine layer of perlite to prevent water evaporation from the substrate. Plants were arranged in double rows with a distance of 35 and 25 cm inter- and intra-rows, respectively, for a density of 11.5 plants m^−2^. Seedlings were irrigated with nutrient solution (NS) provided by a drip irrigation system consisting of a 16 mm polyethylene main pipeline equipped with 2 L h^−1^ drippers. The Hoagland NS had the following composition: 8.0 mM nitrate, 0.7 mM phosphorus, 2.5 mM potassium, 3.0 mM calcium, 1.0 mM sulfur, 0.7 mM magnesium, 1.0 mM ammonium, 1 mM sodium, 1 mM chlorine, 20 μM iron, 9 μM manganese, 0.3 μM cupper, 1.6 μM zinc, 20 μM boron and 0.3 μM molybdenum. The pH and EC of the NS were 6.0 ± 0.2 and 1.2 ± 0.1 dS m^−1^, respectively. Relative humidity and temperature were recorded continuously using WatchDog A150 data loggers (Spectrum Technologies Inc., Aurora, IL, USA; 3%/0.6 °C RH/Temp accuracy) at canopy level at different points of the greenhouse. Climate data were collected at a 30-min interval. Periodic measurements of Photosynthetic Photon Flux Density (PPFD) were recorded from 7:30 AM to 6:30 PM using a handheld spectral radiometer (MSC15, Gigahertz-Optik, Turkenfeld, Germany). Average temperature, relative humidity and PPFD trend recorded during the growing season at the experimental site are shown in Supplementary Figure S1.

### 4.2. Growth, Yield and Sampling

At 25 days after transplanting (DAT), the plants were harvested, weighed for fresh yield determination (g plant^−1^) and separated into leaves and stems. Leaf area was quantified by digital image analysis with ImageJ v1.52a software (U.S. National Institutes of Health, Bethesda, MD, USA). A subsample of leaf tissue was immediately stored at −20 °C for total ascorbic acid and pigment analysis. All harvested tissues were oven-dried at 70 °C until constant weight (~72 h) for dry biomass (g plant^−1^) and leaf dry matter (%) determination. Dried leaves were ground with an MF10.1 cutting-grinding head mill (IKA^®^, Staufen im Breisgau, Baden-Württemberg, Germany) and sieved with MF0.5 sieve (0.5 mm hole size; IKA^®^, Staufen im Breisgau, Baden-Württemberg, Germany) for total nitrogen and minerals determination.

### 4.3. Plant Growth Index and Soil Plant Analysis Development (SPAD) Index

At 8, 14 and 21 DAT on three plants per plot, the plant growth trend was quantified through the growth index (cm^3^ plant^−1^) according to the following equation:

(1) $$\mathrm{GI}=\pi {\left(\frac{D}{2}\right)}^{2}\mathrm{Ht}$$

where D is the width as the average of two perpendicular measurements and Ht is the plant height measured from the soil level to the plant highest point (Supplementary Figure S2).

Contextually, green index (SPAD) measurements were taken on young fully expanded leaves with a handheld Minolta Chlorophyll Meter SPAD-502 (Minolta Camera Co. Ltd., Osaka, Japan). A single average SPAD value for each replicate was obtained by measuring ten leaves per plot.

### 4.4. Leaf Gas Exchange and Maximum Quantum Efficiency of Photosystem II

On July 9 (24 DAT) between 11:00 AM and 2:00 PM, leaf gas exchange measurements and fluorescence emission were performed on healthy fully expanded leaves of three plants per plot. CO_2_ net assimilation rate (A_CO2_; μmol CO_2_ m^−2^ s^−1^), stomatal conductance (gs; mmol H_2_O m^−2^ s^−1^) and transpiration (E; mmol H_2_O m^−2^ s^−1^) were measured using a Li-6400 portable leaf gas exchange analyzer (LI-COR Biosciences, Lincoln, NE, USA). The measurements were performed at ambient CO_2_ concentration and photosynthetic active radiation of 1000 µmol m^−2^ s^−1^, as set in the leaf gas exchange analyzer chamber. Instantaneous water use efficiency (WUEi) was calculated as A_CO2_/E.

On the same date, on 10 min dark-adapted leaves, chlorophyll fluorescence measurements were taken with a portable fluorometer (F_v_/F_m_ Meter, Opti-Sciences Inc., Hudson, NH, USA) on the same leaves used for leaf gas exchange measurements. According to Kitajima and Butler [65], the maximum quantum efficiency of PSII (F_v_/F_m_) was calculated as (F_m_ − F_0_)/F_m_, where F_0_ was the ground signal induced by a blue LED internal light of 1–2 µmol photons m^−2^ s^−1^ and F_m_ was the maximal fluorescence level in the induced darkness by one second of saturating light pulse of 3000 µmol photons m^−2^ s^−1^.

### 4.5. Total Nitrogen and Minerals Determination

Total nitrogen content was determined according to the Kjeldahl method described by Bremner [66]. Briefly, one g of finely ground dry plant sample was mixed with 7 mL of 96% H_2_SO_4_ and 10 mL of 30% (*w/w*) H_2_O_2_, then was mineralized in a DK 20 Heating Digester (Velp^®^ Scientifica, Usmate Velate, Monza Brianza, Italy). The mineralized sample was distilled in a UDK 140 distiller (Velp^®^ Scientifica, Usmate Velate, Monza Brianza, Italy) by adding 33% of NaOH. Ammonia was trapped in H_3_BO_3_ by steam distillation and titrated with 0.1 N H_2_SO_4_. All reagents were purchased from Carlo Erba Reagents Srl (Milan, Italy).

Mineral content in lettuce leaves was determined through ion chromatography (ICS-3000, Thermo Scientific™ Dionex™, Sunnyvale, CA, USA) according to the method described by Rouphael et al. [67]. Briefly, 250 mg of ground dried leaves were extracted in 50 mL of ultrapure water (Arium^®^ Advance EDI pure water system; Sartorius, Goettingen, Lower Saxony, Germany), incubated at 80 °C in a shaking water bath (ShakeTemp SW22, Julabo, Seelbach, Germany) for 10 min, centrifuged at 6000 rpm for 10 min (R-10 M, Remi Elektrotechnik Limited, Mumbai, India) and then filtered by a syringe filter with a 0.45 µm pore size (Whatman International Ltd., Maidstone, Kent, UK). For anions (NO_3_^−^, PO_4_^3–^ and SO_4_^2−^) determination, an IonPac AG11-HC 4 × 50 mm guard column and an IonPac AS11-HC 4 × 250 mm analytical column were used. For cations (K^+^, Ca^2+^, Mg^2+^ and Na^+^) determination, an IonPac CG12A 4 × 250 mm guard column and an IonPac CS12A 4 × 250 mm analytical column were used. All columns were purchased from Thermo Scientific™ Dionex™ (Sunnyvale, CA, USA).

Except nitrate expressed as mg kg^−1^ of fresh weight (FW), all minerals were expressed as mg g^−1^ of dry weight (DW). Total nitrogen was expressed as a percentage (%). Minerals and total nitrogen were analyzed in triplicate.

### 4.6. Morpho-Anatomical Leaf Traits Determination

The LMA was evaluated on nine leaves per treatment as the ratio between leaf DW and leaf area. The number of epidermal cells and stomata were determined on the abaxial and adaxial sides of the same leaves used for leaf gas exchange and LMA measurements, as described by Cirillo et al. [68]. Briefly, leaf impressions were made using cyanoacrylate glue on a microscopy slide. Four images per impression were taken with an optical microscope (Leitz Laborlux 12 microscope, Leica, Wetzlar, Germany) at 20× magnification and were analyzed using ImageJ v1.52a software (U.S. National Institutes of Health, Bethesda, MD, USA) to determine the number of stomata (SN) and epidermal cells (ECN). The following equation was used to calculate the stomatal index expressed as a percentage:

(2) $$\mathrm{Stomatal}\;\mathrm{index}=\frac{\mathrm{SN}}{\mathrm{SN}+\mathrm{ECN}}\times 100$$

Stomatal density and epidermal cell density were calculated as the ratio between the number of cells, and the area photographed for each image (0.241 mm^2^).

### 4.7. Total Ascorbic Acid and Leaf Pigments Determination

Total ascorbic acid determination was performed as described by Kampfenkel et al. [69]. Four hundred milligrams of frozen sample were extracted with 0.8 mL of 6% trichloroacetic acid (TCA). The extract was incubated for 15 min at −20 °C, whereafter 1.2 mL of 6% TCA was added. The homogenate was centrifuged at 4000 rpm for 10 min (R-10 M, Remi Elektrotechnik Limited, Mumbai, India). The absorbance was measured at 525 nm through a UV-Vis spectrophotometer ONDA V-10 Plus (Giorgio Bormac s.r.l, Carpi, Italy).

Pigments (chlorophyll *a*, *b* and carotenoids) of lettuce leaves were determined as described by Wellburn [70]. Briefly, 500 mg of fresh sample was extracted in ammonia acetone, pestled in a ceramic mortar, and centrifuged at 2000 rpm for 10 min (R-10 M, Remi Elektrotechnik Limited, Mumbai, India). Chlorophyll *a*, chlorophyll *b* and carotenoid contents were determined through a UV-Vis spectrophotometer ONDA V-10 Plus (Giorgio Bormac s.r.l, Carpi, Italy) with an absorbance of 647, 664 and 470 nm, respectively.

Chlorophyll a, chlorophyll b, total chlorophylls, carotenoids and total ascorbic acid were expressed as mg 100 g^−1^ DW as suggested by Kováčik [71].

### 4.8. Statistics

The Shapiro–Wilk and Kolmororov–Smirnov procedures were performed to verify that the data had a normal distribution, and the Levene, O’Brien and Bartlet tests were conducted to verify the homogeneity of variances. Data were subjected to two-way analysis of variance (ANOVA) using IBM SPSS Statistics version 20.0 (SPSS Inc., Chicago, Illinois, USA). The mean effect of CV and GIC was compared according to one-way analysis of variance and *t*-Test, respectively. Significant statistical differences were determined by Duncan’s multiple-interval test for the CV × GIC interaction and the CV factor at the level of *p* < 0.05

## 5. Conclusions

**Figure 6:**
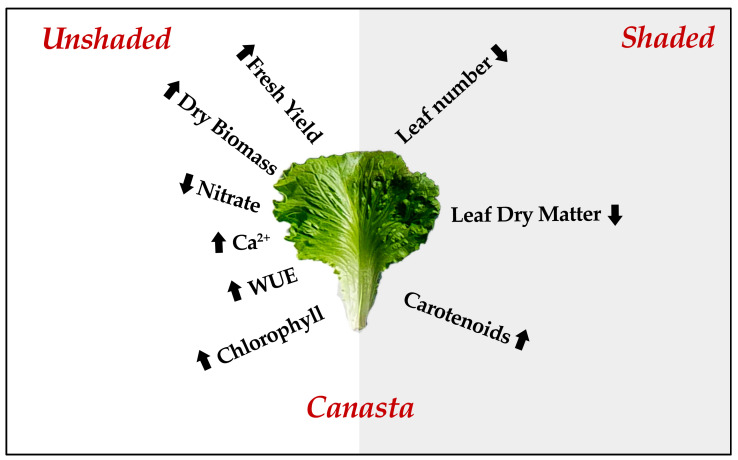
Schematic graphical representation of the productive and adaptive response of *Lactuca sativa* L. cv ‘Canasta’ grown under shaded and unshaded conditions in early summer season.

High light intensity and high temperatures in Mediterranean regions pose a challenge to off-season lettuce production (spring-summer season), affecting growth and yield and resulting in quality losses. In this perspective, the combination of shading and genotypes tolerant to sub-optimal summer conditions is mandatory for off-season lettuce production. Our results showed that different genotypes revealed diverse responses to adverse microclimatic conditions. Among the four genotypes, ‘Canasta’ increased fresh yield and WUE in unshaded treatment (Figure 6). This was correlated to specific morpho-anatomical adaptations of this cultivar, such as reduction of stomatal and epidermal cells density. This highlights the better suitability of ‘Canasta’ to extreme summer conditions, thus presenting it as a promising genotype for off-season production and breeding programs. Nonetheless, the white shading net (49% screening) proved useful in creating an adequate microclimate during the early summer season, ensuring the growth of the more sensitive cultivars ‘Ballerina’, ‘Oak leaf’ and ‘Romaine’. Even though more light has been shed on the adaptive aspects of lettuce grown at high light intensity, future research should be focused on the secondary metabolism response as an additional defense system for plants to adapt to sub-optimal growing conditions successfully.

## Figures and Tables

**Table 1:** Effect of cultivar and greenhouse irradiance conditions on leaf number, leaf area, fresh yield, dry biomass and leaf dry matter in *Lactuca sativa* L.

Source of Variance	Leaf Number	Leaf Area	Fresh Yield	Dry Biomass	Leaf Dry Matter
	(No. plant^−1^)	(cm^2^)	(g plant^−^^1^)	(g plant^−1^)	(%)
Cultivar (CV)					
‘Ballerina’	33.39 ± 0.57 b	3729 ± 119 b	222.7 ± 7.67 c	13.15 ± 0.09 c	5.94 ± 0.22 a
‘Canasta’	30.67 ± 0.61 c	3829 ± 66 b	273.2 ± 5.82 a	13.83 ± 0.54 b	5.05 ± 0.10 b
‘Oak leaf’	31.17 ± 0.61 c	2331 ± 246 c	173.1 ± 9.21 d	8.30 ± 0.16 d	4.84 ± 0.20 c
‘Romaine’	38.67 ± 1.70 a	4204 ± 168 a	241.3 ± 7.08 b	14.59 ± 0.31 a	6.07 ± 0.18 a
	***	***	***	***	***
Greenhouse Irradiance Conditions (GIC)					
Unshaded	34.08 ± 1.48	3269 ± 264	217.7 ± 14.4	12.69 ± 0.85	5.84 ± 0.18
Shaded	32.86 ± 0.69	3777 ± 186	237.4 ± 8.04	12.24 ± 0.68	5.12 ± 0.16
*t*-Test	ns	ns	ns	ns	*
CV × GIC					
‘Ballerina’ × Unshaded	32.33 ± 0.58 c	3472 ± 69 d	206.2 ± 4.02 e	13.24 ± 0.18 b	6.42 ± 0.05 a
‘Ballerina’ × Shaded	34.44 ± 0.40 b	3986 ± 14 b	239.2 ± 2.51 c	13.05 ± 0.03 b	5.46 ± 0.06 bc
‘Canasta’ × Unshaded	31.89 ± 0.11 c	3950 ± 47 bc	285.7 ± 1.80 a	14.97 ± 0.29 a	5.24 ± 0.08 c
‘Canasta’ × Shaded	29.44 ± 0.59 d	3709 ± 72 cd	260.7 ± 3.00 b	12.69 ± 0.25 b	4.87 ± 0.07 d
‘Oak leaf’ × Unshaded	29.89 ± 0.29 d	1802 ± 108 f	152.6 ± 0.83 g	8.04 ± 0.17 c	5.27 ± 0.08 c
‘Oak leaf’ × Shaded	32.44 ± 0.40 c	2860 ± 101 e	193.7 ± 0.50 f	8.57 ± 0.17 c	4.42 ± 0.10 e
‘Romaine’ × Unshaded	42.22 ± 1.31 a	3854 ± 132 bc	226.4 ± 5.05 d	14.53 ± 0.61 a	6.41 ± 0.13 a
‘Romaine’ × Shaded	35.11 ± 0.11 b	4555 ± 6 a	256.2 ± 1.60 b	14.64 ± 0.31 a	5.72 ± 0.14 b
	***	***	***	***	**

Data are mean values ± standard error, *n* = 3. Mean comparisons were performed by Duncan’s Multiple Range Test (DMRT) for CV and by *t*-Test for GIC. Different letters within columns indicate significant mean differences compared by DMRT (*p* = 0.05). ns, *, **, and *** denote nonsignificant or significant effects at *p* ≤ 0.05, 0.01, and 0.001, respectively.

**Table 2:** Effect of cultivar and greenhouse irradiance conditions on total nitrogen and macronutrients accumulation in *Lactuca sativa* L.

Source of Variance	Total N	NO_3_	P	K	Ca	Mg	S	Na
	(%)	(mg kg^−1^ FW)	(mg g^−1^ DW)	(mg g^−1^ DW)	(mg g^−1^ DW)	(mg g^−1^ DW)	(mg g^−1^ DW)	(mg g^−1^ DW)
Cultivar (CV)								
‘Ballerina’	3.32 ± 0.10 c	2017 ± 69 b	4.24 ± 0.17 c	41.25 ± 0.66 c	11.11 ± 0.77 a	3.68 ± 0.09 b	1.48 ± 0.13 a	2.11 ± 0.07 b
‘Canasta’	3.90 ± 0.10 a	2279 ± 69 a	5.03 ± 0.41 a	39.17 ± 0.64 d	8.27 ± 1.14 b	3.22 ± 0.19 c	1.63 ± 0.09 a	1.40 ± 0.12 c
‘Oak leaf’	3.65 ± 0.12 b	2214 ± 50 a	4.74 ± 0.19 b	52.56 ± 3.31 a	10.40 ± 0.42 a	3.16 ± 0.28 c	1.25 ± 0.06 b	2.07 ± 0.11 b
‘Romaine’	3.40 ± 0.03 c	1744 ± 39 c	4.19 ± 0.12 c	45.45 ± 1.37 b	10.80 ± 0.60 a	4.22 ± 0.16 a	1.08 ± 0.07 b	3.57 ± 0.14 a
	***	***	***	***	***	***	***	***
Greenhouse Irradiance Conditions (GIC)								
Unshaded	3.41 ± 0.07	1963 ± 56	4.07 ± 0.07	41.71 ± 0.86	11.66 ± 0.27	3.40 ± 0.17	1.50 ± 0.09	2.30 ± 0.21
Shaded	3.72 ± 0.09	2165 ± 77	5.03 ± 0.18	47.50 ± 2.37	8.63 ± 0.53	3.74 ± 0.18	1.21 ± 0.07	2.27 ± 0.28
*t*-Test	*	*	***	*	***	ns	*	ns
CV × GIC								
‘Ballerina’ × Unshaded	3.12 ± 0.06 d	1877 ± 24 c	3.87 ± 0.11 e	40.33 ± 1.15 def	12.78 ± 0.28 a	3.56 ± 0.05 b	1.74 ± 0.11 a	2.25 ± 0.02 b
‘Ballerina’ × Shaded	3.53 ± 0.06 c	2156 ± 60 b	4.61 ± 0.07 c	42.16 ± 0.18 de	9.45 ± 0.38 c	3.79 ± 0.15 b	1.21 ± 0.06 b	1.98 ± 0.05 bc
‘Canasta’ × Unshaded	3.72 ± 0.10 b	2143 ± 60 b	4.13 ± 0.01 de	38.64 ± 0.89 f	10.70 ± 0.26 bc	3.45 ± 0.28 bc	1.80 ± 0.06 a	1.67 ± 0.07 c
‘Canasta’ × Shaded	4.08 ± 0.06 a	2416 ± 39 a	5.93 ± 0.16 a	39.69 ± 1.00 ef	5.83 ± 0.67 d	2.98 ± 0.20 cd	1.45 ± 0.11 b	1.14 ± 0.02 d
‘Oak leaf’ × Unshaded	3.40 ± 0.05 c	2104 ± 13 b	4.33 ± 0.13 cd	45.29 ± 1.27 c	11.20 ± 0.28 b	2.59 ± 0.17 d	1.24 ± 0.08 b	1.85 ± 0.07 c
‘Oak leaf’ × Shaded	3.90 ± 0.05 ab	2324 ± 4 a	5.14 ± 0.07 b	59.84 ± 0.38 a	9.60 ± 0.39 c	3.73 ± 0.17 b	1.26 ± 0.12 b	2.29 ± 0.09 b
‘Romaine’ × Unshaded	3.41 ± 0.06 c	1727 ± 82 d	3.94 ± 0.06 e	42.58 ± 0.52 d	11.96 ± 0.41 ab	3.98 ± 0.10 ab	1.22 ± 0.06 b	3.44 ± 0.19 a
‘Romaine’ × Shaded	3.38 ± 0.02 c	1762 ± 22 cd	4.44 ± 0.07 c	48.32 ± 0.95 b	9.64 ± 0.51 c	4.47 ± 0.24 a	0.94 ± 0.05 c	3.69 ± 0.20 a
	**	*	***	***	**	**	*	**

Data are mean values ± standard error, *n* = 3. Mean comparisons were performed by Duncan’s Multiple Range Test (DMRT) for CV and by *t*-Test for GIC. Different letters within columns indicate significant mean differences compared by DMRT (*p* = 0.05). ns, *, **, and *** denote nonsignificant or significant effects at *p* ≤ 0.05, 0.01, and 0.001, respectively.

**Table 3:** Effect of cultivar and greenhouse irradiance conditions on SPAD index, fluorescence (F_v_/F_m_ ratio) and leaf mass area (LMA) in *Lactuca sativa* L.

Source of Variance	SPAD			Fluorescence	LMA
	8 DAT	14 DAT	21 DAT	F_v_/F_m_ Ratio	(g DW m^−2^)
Cultivar (CV)					
‘Ballerina’	27.73 ± 0.85 a	31.81 ± 0.28 b	35.51 ± 0.59 a	0.79 ± 0.02 ab	47.88 ± 1.06 a
‘Canasta’	27.53 ± 1.09 a	31.97 ± 0.70 b	35.82 ± 0.14 a	0.79 ± 0.02 a	37.63 ± 1.76 c
‘Oak leaf’	20.79 ± 0.73 b	22.85 ± 0.57 c	22.47 ± 1.07 c	0.78 ± 0.02 b	26.19 ± 2.20 d
‘Romaine’	28.23 ± 0.53 a	33.40 ± 0.38 a	34.05 ± 0.35 b	0.80 ± 0.02 a	42.60 ± 1.90 b
	***	***	***	*	***
Greenhouse Irradiance Conditions (GIC)					
Unshaded	27.77 ± 0.97	30.88 ± 1.21	32.22 ± 1.31	0.75 ± 0.00	42.01 ± 2.32
Shaded	24.37 ± 0.93	29.13 ± 1.34	31.70 ± 2.05	0.83 ± 0.00	35.14 ± 2.65
*t*-Test	*	ns	ns	***	***
CV × GIC					
‘Ballerina’ × Unshaded	29.52 ± 0.52 a	32.34 ± 0.21 bc	34.25 ± 0.29 c	0.74 ± 0.00	49.88 ± 1.22
‘Ballerina’ × Shaded	25.94 ± 0.34 c	31.28 ± 0.28 cd	36.77 ± 0.22 a	0.84 ± 0.01	45.87 ± 0.34
‘Canasta’ × Unshaded	29.88 ± 0.47 a	33.40 ± 0.37 ab	35.60 ± 0.18 b	0.76 ± 0.01	41.47 ± 0.53
‘Canasta’ × Shaded	25.18 ± 0.41 c	30.54 ± 0.54 d	36.05 ± 0.09 ab	0.83 ± 0.01	33.79 ± 0.72
‘Oak leaf’ × Unshaded	22.35 ± 0.45 d	24.04 ± 0.23 e	24.82 ± 0.38 d	0.73 ± 0.01	30.48 ± 2.37
‘Oak leaf’ × Shaded	19.23 ± 0.25 e	21.66 ± 0.43 f	20.12 ± 0.20 e	0.82 ± 0.00	21.91 ± 0.36
‘Romaine’ × Unshaded	29.33 ± 0.38 a	33.74 ± 0.69 a	34.22 ± 0.52 c	0.76 ± 0.01	46.21 ± 2.14
‘Romaine’ × Shaded	27.12 ± 0.17 b	33.06 ± 0.38 ab	33.88 ± 0.55 c	0.84 ± 0.00	38.99 ± 0.67
	*	*	***	ns	ns

Data are mean values ± standard error, *n* = 3. Mean comparisons were performed by Duncan’s Multiple Range Test (DMRT) for CV and by *t*-Test for GIC. Different letters within columns indicate significant differences compared by DMRT (*p* = 0.05). ns, *, and *** denote nonsignificant or significant effects at *p* ≤ 0.05 and 0.001, respectively.

**Table 4:** Effect of cultivar and greenhouse irradiance conditions on total ascorbic acid (TAA) and leaf pigments accumulation in *Lactuca sativa* L.

Source of Variance	TAA	Chlorophyll a	Chlorophyll b	Total Chlorophylls	Carotenoids
	(mg g^−1^ DW)	(mg g^−1^ DW)	(mg g^−1^ DW)	(mg g^−1^ DW)	(mg g^−1^ DW)
Cultivar (CV)					
‘Ballerina’	10.02 ± 0.72 b	15.06 ± 0.50 ab	9.17 ± 0.25 a	24.23 ± 0.75 a	5.98 ± 0.35 b
‘Canasta’	13.67 ± 0.47 a	15.61 ± 0.42 a	9.09 ± 0.69 a	24.70 ± 1.06 a	7.07 ± 0.25 a
‘Oak leaf’	7.39 ± 0.71 c	13.97 ± 0.32 b	8.09 ± 0.28 b	22.06 ± 0.58 b	7.01 ± 0.16 a
‘Romaine’	6.25 ± 0.52 c	11.74 ± 0.23 c	6.67 ± 0.20 c	18.41 ± 0.37 c	5.99 ± 0.14 b
	***	***	***	***	***
Greenhouse Irradiance Conditions (GIC)					
Unshaded	9.25 ± 0.66	14.03 ± 0.52	8.61 ± 0.42	22.64 ± 0.93	6.07 ± 0.19
Shaded	9.42 ± 1.18	14.16 ± 0.51	7.90 ± 0.36	22.06 ± 0.85	6.96 ± 0.18
*t*-Test	ns	ns	ns	ns	**
CV × GIC					
‘Ballerina’ × Unshaded	8.49 ± 0.28 c	13.96 ± 0.16 b	8.64 ± 0.19 bc	22.60 ± 0.34 b	5.21 ± 0.10 e
‘Ballerina’ × Shaded	11.56 ± 0.39 b	16.15 ± 0.18 a	9.70 ± 0.04 ab	25.85 ± 0.22 a	6.74 ± 0.07 bc
‘Canasta’ × Unshaded	12.81 ± 0.29 ab	16.39 ± 0.48 a	10.57 ± 0.36 a	26.96 ± 0.69 a	6.58 ± 0.18 c
‘Canasta’ × Shaded	14.52 ± 0.54 a	14.84 ± 0.23 ab	7.61 ± 0.19 cd	22.45 ± 0.28 b	7.57 ± 0.22 a
‘Oak leaf’ × Unshaded	8.65 ± 0.41 c	13.99 ± 0.60 b	8.40 ± 0.44 bc	22.39 ± 1.03 b	6.68 ± 0.06 bc
‘Oak leaf’ × Shaded	6.13 ± 0.87 cd	13.94 ± 0.39 b	7.79 ± 0.31 cd	21.73 ± 0.70 b	7.34 ± 0.14 ab
‘Romaine’ × Unshaded	7.04 ± 0.10 cd	11.78 ± 0.23 c	6.84 ± 0.21 d	18.61 ± 0.02 c	5.80 ± 0.18 de
‘Romaine’ × Shaded	5.46 ± 0.84 d	11.71 ± 0.46 c	6.49 ± 0.35 d	18.21 ± 0.81 c	6.18 ± 0.16 cd
	***	**	***	***	*

Data are mean values ± standard error, *n* = 3. Mean comparisons were performed by Duncan’s Multiple Range Test (DMRT) for CV and by *t*-Test for GIC. Different letters within columns indicate significant mean differences compared by DMRT (*p* = 0.05). ns, *, **, and *** denote nonsignificant or significant effects at *p* ≤ 0.05, 0.01, and 0.001, respectively.

## Data Availability

The datasets generated for this study are available on request to the corresponding author.

## Supplementary Materials

The following are available online at https://www.mdpi.com/article/10.3390/plants10061179/s1, Figure S1: Photosynthetic Photon Flux Density (PPFD), average air temperature and air relative humidity recorded during the growing season at the experimental site under shaded and unshaded conditions. Figure S2: Interaction between Cultivar (CV) and Greenhouse Irradiance Conditions (GIC) on plant growth trend quantified through growth index (cm^3^ plant^-1^) at different days after transplant. Data are mean values ± standard error, *n* = 3. Mean comparisons were performed by Duncan’s Multiple Range Test (DMRT) for CV and by *t*-Test for GIC. Different letters within columns indicate significant mean differences. ** and *** denote significant effects at *p* ≤ 0.01 and 0.001, respectively.

## References

[1] Pérez-López U., Sgherri C., Miranda-Apodaca J., Micaelli F., Lacuesta M., Mena-Petite A., Quartacci M.F., Muñoz-Rueda A. (2018). Concentration of phenolic compounds is increased in lettuce grown under high light intensity and elevated CO_2_. Plant Physiol. Biochem..

[2] Kosma C., Triantafyllidis V., Papasavvas A., Salahas G., Patakas A. (2013). Yield and nutritional quality of greenhouse lettuce as affected by shading and cultivation season. Emir. J. Food Agric..

[3] USDA (United State Departement of Agricolture). https://fdc.nal.usda.gov/fdc-app.html#/food-details/169247/nutrients.

[4] Kim M.J., Moon Y., Tou J.C., Mou B., Waterland N.L. (2016). Nutritional value, bioactive compounds and health benefits of lettuce (*Lactuca sativa* L.). J. Food Compos. Anal..

[5] Fu W., Li P., Wu Y. (2012). Effects of different light intensities on chlorophyll fluorescence characteristics and yield in lettuce. Sci. Hortic..

[6] Hunter B.L. (2010). Enhancing Out-of-Season Production of Tomatoes and Lettuce Using High Tunnels.

[7] Ilić Z.S., Milenković L., Stanojević L., Cvetković D., Fallik E. (2012). Effects of the modification of light intensity by color shade nets on yield and quality of tomato fruits. Sci. Hortic..

[8] Meena R.K., Vashisth A., Singh R., Singh B., Manjaih K.M. (2014). Study on change in microenvironment under different colour shade nets and its impact on yield of spinach (*Spinacia oleracea* L.). J. Agrometeorol..

[9] Flaishman M.A., Peles Y., Dahan Y., Milo-Cochavi S., Frieman A., Naor A. (2015). Differential response of cell-cycle and cell-expansion regulators to heat stress in apple (*Malus domestica*) fruitlets. Plant Sci..

[10] Ilić S.Z., Milenković L., Dimitrijević A., Stanojević L., Cvetković D., Kevrešan F.E., Mastilović J. (2017). Light modification by color nets improve quality of lettuce from summer production. Sci. Hortic..

[11] Lafta A., Sandoya G., Mou B. (2020). Genetic Variation and Genotype by Environment Interaction for Heat Tolerance in Crisphead Lettuce. HortScience.

[12] Al-Said F., Hadley P., Pearson S., Khan M.M., Iqbal Q. (2018). Effect of high temperature and exposure duration on stem elongation of iceberg lettuce. Pak. J. Agric. Sci..

[13] Yan Z., Ma T., Guo S., Liu R., Li M. (2021). Leaf anatomy, photosynthesis and chlorophyll fluorescence of lettuce as influenced by arbuscular mycorrhizal fungi under high temperature stress. Sci. Hortic..

[14] Formisano L., El-Nakhel C., Corrado G., De Pascale S., Rouphael Y. (2020). Biochemical, physiological, and productive response of greenhouse vegetables to suboptimal growth environment induced by insect nets. Biology.

[15] Carotti L., Graamans L., Puksic F., Butturini M., Meinen E., Heuvelink E., Stanghellini C. (2021). Plant Factories Are Heating Up: Hunting for the Best Combination of Light Intensity, Air Temperature and Root-Zone Temperature in Lettuce Production. Front. Plant Sci..

[16] Briassoulis D., Mistriotis A., Eleftherakis D. (2007). Mechanical behaviour and properties of agricultural nets. Part II: Analysis of the performance of the main categories of agricultural nets. Polym. Test..

[17] Shahak Y. (2014). Photoselective netting: An overview of the concept, r and d and practical implementation in agriculture. Acta Hortic..

[18] Semida W.M., Ammar M.S., El-Sawah N.A. (2017). Effects of Shade Level and Microenvironment on Vegetative Growth, Physiological and Biochemical Characteristics of Transplanted Cucumber (*Cucumis sativus*). Arch. Agric. Environ. Sci..

[19] Fu W., Li P., Wu Y., Tang J. (2012). Effects of different light intensities on anti-oxidative enzyme activity, quality and biomass in lettuce. Hortic. Sci..

[20] Ilić Z.S., Milenković L., Šunić L., Barać S., Mastilović J., Kevrešan Ž., Fallik E. (2017). Effect of shading by coloured nets on yield and fruit quality of sweet pepper. Zemdirb. Agric..

[21] Roeber V.M., Bajaj I., Rohde M., Schmülling T., Cortleven A. (2020). Light acts as a stressor and influences abiotic and biotic stress responses in plants. Plant. Cell Environ..

[22] Takahashi S., Badger M.R. (2011). Photoprotection in plants: A new light on photosystem II damage. Trends Plant Sci..

[23] Lichtenthaler H.K., Burkart S. (1999). Photosynthesis and high light stress. Wild.

[24] Szymańska R., Ślesak I., Orzechowska A., Kruk J. (2017). Physiological and biochemical responses to high light and temperature stress in plants. Environ. Exp. Bot..

[25] Ruban A.V. (2009). Plants in light. Commun. Integr. Biol..

[26] Banaś A.K., Aggarwal C., Łabuz J., Sztatelman O., Gabryś H. (2012). Blue light signalling in chloroplast movements. J. Exp. Bot..

[27] O’Carrigan A., Hinde E., Lu N., Xu X.Q., Duan H., Huang G., Mak M., Bellotti B., Chen Z.H. (2014). Effects of light irradiance on stomatal regulation and growth of tomato. Environ. Exp. Bot..

[28] Poorter H., Niinemets Ü., Poorter L., Wright I.J., Villar R. (2009). Causes and consequences of variation in leaf mass per area (LMA): A meta-analysis. New Phytol..

[29] Zha L., Liu W., Zhang Y., Zhou C., Shao M. (2019). Morphological and Physiological Stress Responses of Lettuce to Different Intensities of Continuous Light. Front. Plant Sci..

[30] Oguchi R., Onoda Y., Terashima I., Tholen D., Adams W.W., Terashima I. (2018). Leaf Anatomy and Function. The Leaf: A Platform for Performing Photosynthesis.

[31] Bartoli G., Bottega S., Spano C. (2015). Morpho-anatomical and physiological traits of Agrostis castellana living in an active geothermal alteration field. Biology.

[32] Schymanski S.J., Or D., Zwieniecki M. (2013). Stomatal Control and Leaf Thermal and Hydraulic Capacitances under Rapid Environmental Fluctuations. PLoS ONE.

[33] Wang C., He J., Zhao T.H., Cao Y., Wang G., Sun B., Yan X., Guo W., Li M.H. (2019). The smaller the leaf is, the faster the leaf water loses in a temperate forest. Front. Plant Sci..

[34] Wang J., Lu W., Tong Y., Yang Q. (2016). Leaf morphology, photosynthetic performance, chlorophyll fluorescence, stomatal development of lettuce (*Lactuca sativa* L.) exposed to different ratios of red light to blue light. Front. Plant Sci..

[35] Franks P.J., Doheny-Adams T.W., Britton-Harper Z.J., Gray J.E. (2015). Increasing water-use efficiency directly through genetic manipulation of stomatal density. New Phytol..

[36] Pathare V.S., Koteyeva N., Cousins A.B. (2020). Increased adaxial stomatal density is associated with greater mesophyll surface area exposed to intercellular air spaces and mesophyll conductance in diverse C4 grasses. New Phytol..

[37] Harrison E.L., Arce Cubas L., Gray J.E., Hepworth C. (2020). The influence of stomatal morphology and distribution on photosynthetic gas exchange. Plant J..

[38] Casson S., Gray J.E. (2008). Influence of environmental factors on stomatal development. New Phytol..

[39] Crawford A.J., McLachlan D.H., Hetherington A.M., Franklin K.A. (2012). High temperature exposure increases plant cooling capacity. Curr. Biol..

[40] Quint M., Delker C., Franklin K.A., Wigge P.A., Halliday K.J., Van Zanten M. (2016). Molecular and genetic control of plant thermomorphogenesis. Nat. Plants.

[41] Chaves M.M., Costa J.M., Zarrouk O., Pinheiro C., Lopes C.M., Pereira J.S. (2016). Controlling stomatal aperture in semi-arid regions—The dilemma of saving water or being cool?. Plant Sci..

[42] Muir C.D. (2017). Light and growth form interact to shape stomatal ratio among British angiosperms. New Phytol..

[43] Katerji N., Mastrorilli M., Rana G. (2008). Water use efficiency of crops cultivated in the Mediterranean region: Review and analysis. Eur. J. Agron..

[44] Tabit Shaban N., Tzvetkova N., Cherkez R., Parvanova P. (2016). Evaluation of response of lettuce (*Lactuca sativa* L.) to temperature and light stress. Acta Agrobot..

[45] Lourdes R., Hipol B., Dionisio-Sese M.L. (2014). Impact of Light Variation on the Antioxidant Properties of Red Lettuce. Electron. J. Biol..

[46] Trojak M., Skowron E. (2017). Role of anthocyanins in high-light stress response. World Sci. News.

[47] Hassanien R.H.E., Li M. (2017). Influences of greenhouse-integrated semi-transparent photovoltaics on microclimate and lettuce growth. Int. J. Agric. Biol. Eng..

[48] Araki Y., Inoue S., Murakami K., Lee J.-M., Watada A.E., Gross K.C., Lee S.-K. (1999). Effect of shading on growth and quality of summer spinach. Proceedings of the Acta Horticulturae.

[49] Stagnari F., Galieni A., Pisante M. (2015). Shading and nitrogen management affect quality, safety and yield of greenhouse-grown leaf lettuce. Sci. Hortic..

[50] Wolff X.Y., Coltman R.R. (2019). Productivity Under Shade in Hawaii of Five Crops Grown as Vegetables in the Tropics. J. Am. Soc. Hortic. Sci..

[51] Lake J.A., Quick W.P., Beerling D.J., Woodward F.I. (2001). Signals from mature to new leaves. Nature.

[52] Coupe S.A., Palmer B.G., Lake J.A., Overy S.A., Oxborough K., Woodward F.I., Gray J.E., Quick W.P. (2006). Systemic signalling of environmental cues in Arabidopsis leaves. J. Exp. Bot..

[53] Miyazawa S.I., Livingston N.J., Turpin D.H. (2006). Stomatal development in new leaves is related to the stomatal conductance of mature leaves in poplar (*Populus trichocarpa × P. deltoides*). J. Exp. Bot..

[54] Bertolino L.T., Caine R.S., Gray J.E. (2019). Impact of stomatal density and morphology on water-use efficiency in a changing world. Front. Plant Sci..

[55] Carins Murphy M.R., Jordan G.J., Brodribb T.J. (2016). Cell expansion not cell differentiation predominantly co-ordinates veins and stomata within and among herbs and woody angiosperms grown under sun and shade. Ann. Bot..

[56] Zhou Y.H., Zhang Y.Y., Zhao X., Yu H.J., Shi K., Yu J.Q. (2009). Impact of light variation on development of photoprotection, antioxidants, and nutritional value in *Lactuca sativa* L.. J. Agric. Food Chem..

[57] Gerganova M., Popova A.V., Stanoeva D., Velitchkova M. (2016). Tomato plants acclimate better to elevated temperature and high light than to treatment with each factor separately. Plant Physiol. Biochem..

[58] Ilić Z.S., Milenković L., Šunić L., Manojlović M. (2018). Color Shade Nets Improve Vegetables Quality at Harvest and Maintain Quality during Storage. Contemp. Agric..

[59] Rouphael Y., Petropoulos S.A., El-Nakhel C., Pannico A., Kyriacou M.C., Giordano M., Troise A.D., Vitaglione P., De Pascale S. (2019). Reducing Energy Requirements in Future Bioregenerative Life Support Systems (BLSSs): Performance and Bioactive Composition of Diverse Lettuce Genotypes Grown Under Optimal and Suboptimal Light Conditions. Front. Plant. Sci..

[60] Colla G., Kim H.J., Kyriacou M.C., Rouphael Y. (2018). Nitrate in fruits and vegetables. Sci. Hortic..

[61] Reinink K., Groenwold R., Bootsma A. (1987). Genotypical differences in nitrate content in *Lactuca sativa* L. and related species and correlation with dry matter content. Euphytica.

[62] Malhotra H., Vandana, Sharma S., Pandey R., Hasanuzzaman M., Fujita M., Oku H., Nahar K., Hawrylak-Nowak B. (2018). Phosphorus nutrition: Plant growth in response to deficiency and excess. Plant Nutrients and Abiotic Stress Tolerance.

[63] Rychter A.M., Rao I.M. (2005). Role of phosphorus in photosynthetic carbon metabolism. Handbook of Photosynthesis.

[64] Pathak J., Ahmed H., Kumari N., Pandey A., Rajneesh, Sinha R.P. (2020). Role of Calcium and Potassium in Amelioration of Environmental Stress in Plants. Prot. Chem. Agents Amelior. Plant Abiotic Stress.

[65] Kitajima M., Butler W.L. (1975). Excitation spectra for Photosystem I and Photosystem II in chloroplasts and the spectral characteristics of the distribution of quanta between the two photosystems. BBA Bioenerg..

[66] Bremner J.M., Black C.A., Evans D., White J.L., Ensminger L.E., Clark F.E. (1965). Total nitrogen. Methods of Soil Analysis. Part 2. CHemical and Microbiological Properties. Agronomy Monograph 9.

[67] Rouphael Y., Colla G., Giordano M., El-Nakhel C., Kyriacou M.C., De Pascale S. (2017). Foliar applications of a legume-derived protein hydrolysate elicit dose-dependent increases of growth, leaf mineral composition, yield and fruit quality in two greenhouse tomato cultivars. Sci. Hortic..

[68] Cirillo V., D’Amelia V., Esposito M., Amitrano C., Carillo P., Carputo D., Maggio A. (2021). Anthocyanins are Key Regulators of Drought Stress Tolerance in Tobacco. Biology.

[69] Kampfenkel K., Van Montagu M., Inzé D. (1995). Extraction and determination of ascorbate and dehydroascorbate from plant tissue. Anal. Biochem..

[70] Wellburn A.R. (1994). The Spectral Determination of Chlorophylls a and b, as well as Total Carotenoids, Using Various Solvents with Spectrophotometers of Different Resolution. J. Plant Physiol..

[71] Kováčik J. (2021). Basic physiology and biochemistry in environmental/experimental plant studies: How to quantify and interpret metabolites correctly. Environ. Pollut..

