# Supplementary material for: Divergent Leaf Morpho-Physiological and Anatomical Adaptations of Four Lettuce Cultivars in Response to Different Greenhouse Irradiance Levels in Early Summer Season
Source: Plants (Basel). 2021 Jun 9;10(6):1179. doi: 10.3390/plants10061179 (PMC8226882; doi:10.3390/plants10061179)
Supplement: Supplementary file 1 [file plants-10-01179-s001.zip › plants-1247377-supplementary.pdf]

plants

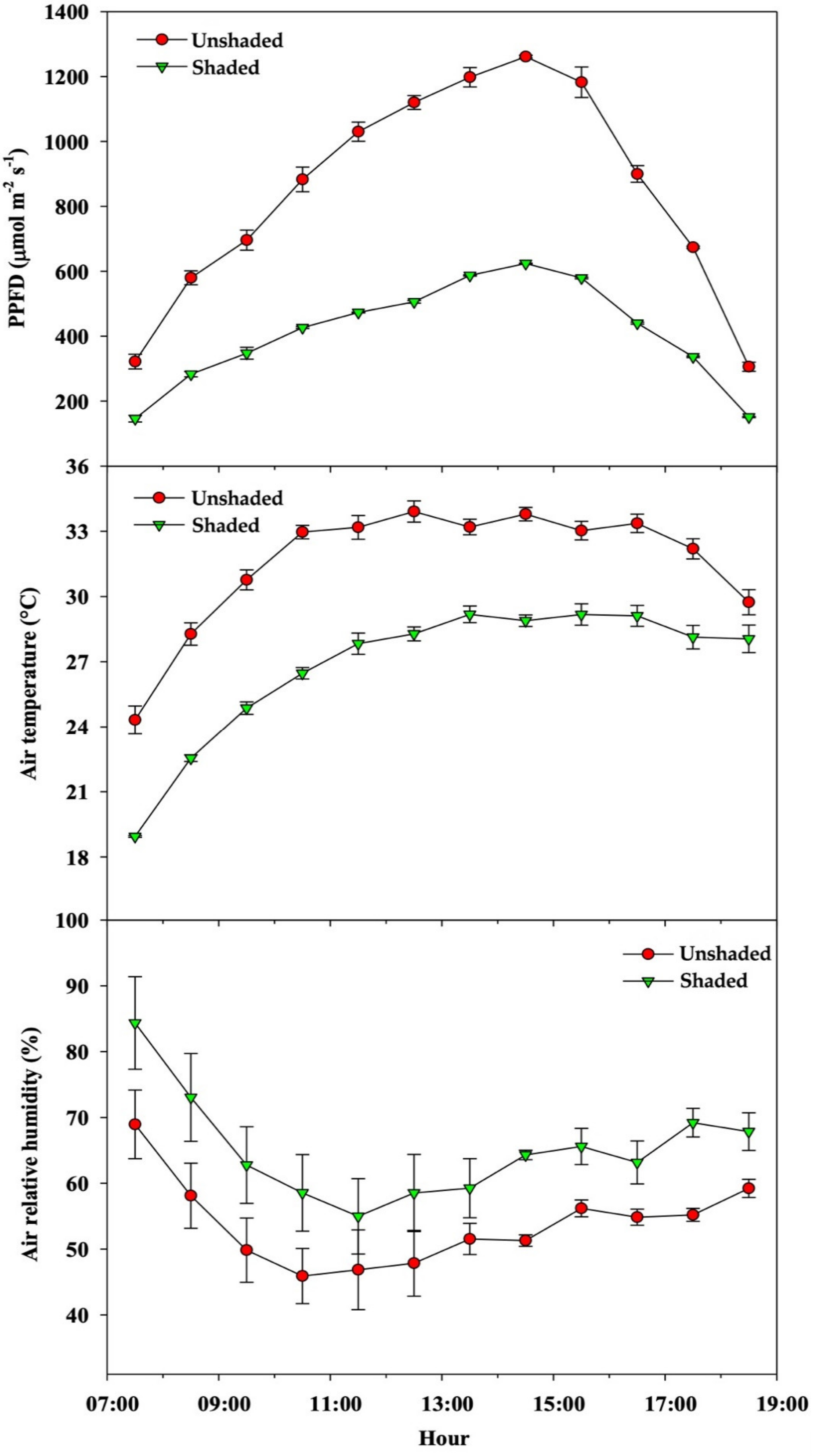

Figure S1. Photosynthetic Photon Flux Density (PPFD), average air temperature, and air relative humidity recorded during the growing season at the experimental site under shaded and unshaded conditions.

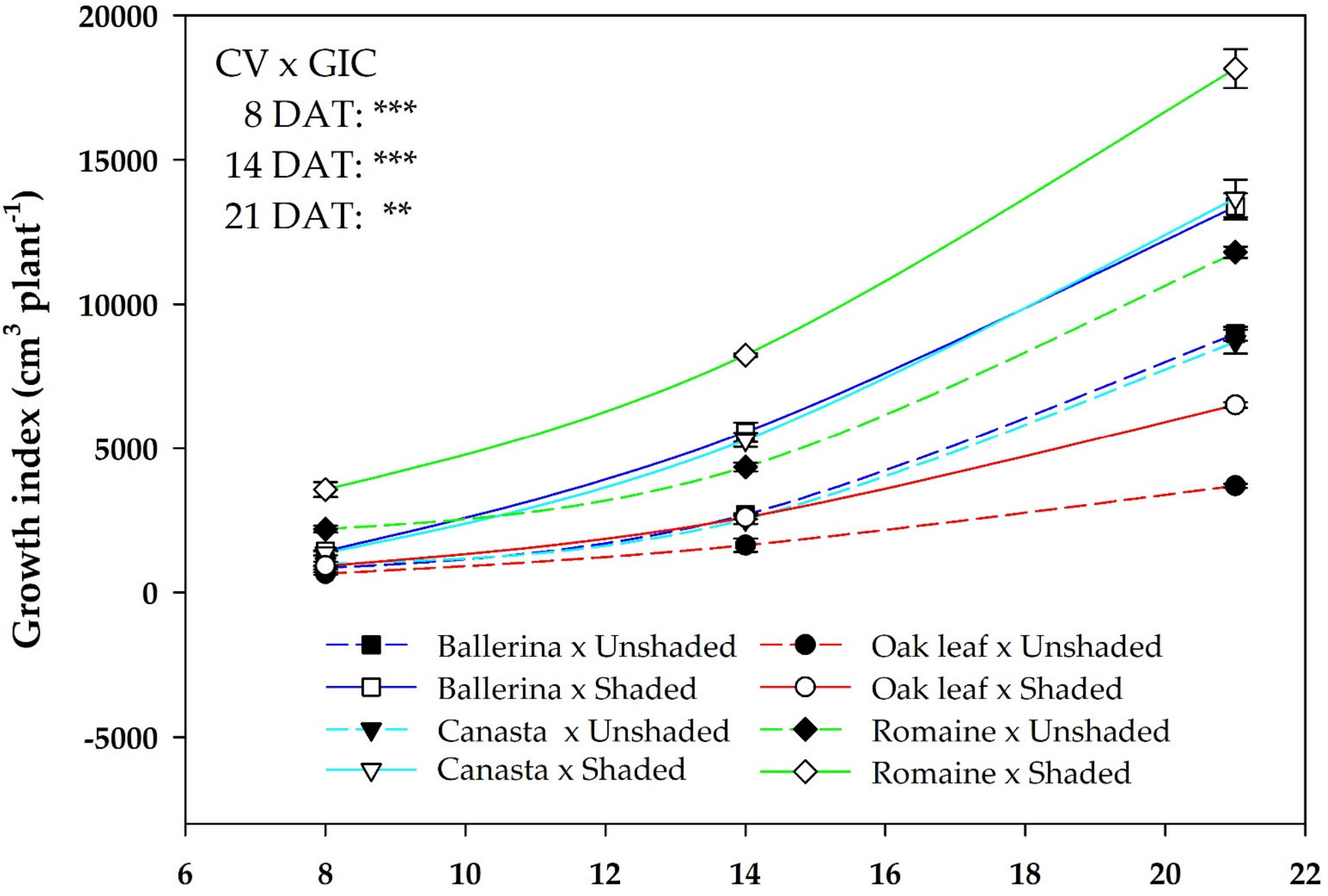

Figure S2. Interaction between Cultivar (CV) and Greenhouse Light Conditions (GLC) on plant growth trend quantified through growth index (cm^3 $plant^{-1}$) at different days after transplant. Data are mean values ± standard error, n = 3. Mean comparisons were performed by Duncan's Multiple Range Test for CV and by t-Test for GLC. Different letters within columns indicate significant mean differences. ** and *** denote significant effects at $p \leq 0.01$ and 0.001, respectively.
